# The effect of self-administered methamphetamine on GABAergic interneuron populations and functional connectivity of the nucleus accumbens and prefrontal cortex

**DOI:** 10.1007/s00213-022-06175-9

**Published:** 2022-08-03

**Authors:** Katherine J. Robinson, Nicholas A. Everett, Sarah J. Baracz, Jennifer L. Cornish

**Affiliations:** 1 Department of Psychology, Faculty of Human Sciences, Macquarie University, Sydney, NSW Australia; 2 School of Psychology, Faculty of Sciences, The University of Sydney, Sydney, NSW Australia

**Keywords:** Methamphetamine, Drugs of abuse, Self-administration, GABAergic interneurons, Neuronal nitric oxide synthase, Parvalbumin, Calretinin, Relapse to methamphetamine, Methamphetamine-induced neuroadaptations

## Abstract

**Introduction:**

Methamphetamine (METH, “ice”) is a potent and addictive psychostimulant. Abuse of METH perturbs neurotransmitter systems and induces neurotoxicity; however, the neurobiological mechanisms which underlie addiction to METH are not fully understood, limiting the efficacy of available treatments. Here we investigate METH-induced changes to neuronal nitric oxide synthase (nNOS), parvalbumin and calretinin-expressing GABAergic interneuron populations within the nucleus accumbens (NAc), prefrontal cortex (PFC) and orbitofrontal cortex (OFC). We hypothesise that dysfunction or loss of these GABAergic interneuron populations may disrupt the excitatory/inhibitory balance within the brain.

**Methods:**

Male Long Evans rats (*N* = 32) were trained to lever press for intravenous METH or received yoked saline infusions. Following 14 days of behavioural extinction, animals were given a non-contingent injection of saline or METH (1 mg/kg, IP) to examine drug-primed reinstatement to METH-seeking behaviours. Ninety minutes post-IP injection, animals were culled and brain sections were analysed for Fos, nNOS, parvalbumin and calretinin immunoreactivity in eight distinct subregions of the NAc, PFC and OFC.

**Results:**

METH exposure differentially affected GABAergic populations, with METH self-administration increasing nNOS immunoreactivity at distinct locations in the prelimbic cortex and decreasing parvalbumin immunoreactivity in the NAc. METH self-administration triggered reduced calretinin immunoreactivity, whilst acute METH administration produced a significant increase in calretinin immunoreactivity. As expected, non-contingent METH-priming treatment increased Fos immunoreactivity in subregions of the NAc and PFC.

**Conclusion:**

Here we report that METH exposure in this model may alter the function of GABAergic interneurons in more subtle ways, such as alterations in neuronal firing or synaptic connectivity.

**Supplementary Information:**

The online version contains supplementary material available at 10.1007/s00213-022-06175-9.

## Introduction

Methamphetamine (METH or “ice”) is a highly potent psychostimulant drug and is considered one of the most harmful illicit drugs, detrimentally impacting individual drug users and society more broadly (Courtney & Ray, 2014; Morley et al., 2017; Panenka et al., 2013). Administration of METH has been shown to perturb neurotransmitter systems within the brain (Cruickshank & Dyer, 2009), activating the mesocorticolimbic dopaminergic reward circuit and triggering supraphysiologic release of monoamines (Hyman & Malenka, 2001; Hyman et al., 2006). Repeated or chronic administration of METH can induce persisting neurotoxic effects, including accumulation of reactive oxygen species and oxidative stress within dopaminergic neurons (Courtney & Ray, 2014; De Vito & Wagner, 1989; Fricks-Gleason & Keefe, 2013). These drug-induced neuroadaptations persist despite prolonged periods of abstinence or cessation of drug use (Cruickshank & Dyer, 2009; Di Chiara, 2002; Kelley & Berridge, 2002). Presently, our understanding of the neurobiological mechanisms that lead to METH use disorder remains limited (Van den Oever et al., 2010). Unfortunately, current available treatments for METH abuse, including cognitive behavioural therapy, are ineffective in preventing relapse (Morley et al., 2017); there are no approved or effective pharmacotherapies for METH addiction in any jurisdiction globally. Novel pharmacotherapies are needed, but hindered by a lack of understanding of the neurobiology of METH use disorder.

There is evidence that the function of key substrates within the mesocorticolimbic dopaminergic reward circuit, such as the nucleus accumbens (NAc) and prefrontal cortex (PFC), may be disrupted following repeated exposure to addictive drugs (Moorman et al., 2015) and that this disrupted function is causally driving addiction. However, there is a distinct lack of research examining METH-induced activation of specific neuronal populations within these regions and the resulting impact on overall circuit function. It has been suggested that increased dopamine release in the NAc may act to increase glutamatergic activity within the PFC (Hyman & Malenka, 2001; Jackson & Moghaddam, 2001; Kelley & Berridge, 2002; Yager et al., 2015) and that increased activity of glutamatergic PFC-NAc projections may drive drug-induced neuroadaptations (Hyman & Malenka, 2001; Kearns et al., 2018; McFarland et al., 2003; Scofield et al., 2016; Yager et al., 2015). However, METH could also act to disrupt the function of γ-amino-butyric acid (GABA) (Miller & Marshall, 2004; Morshedi & Meredith, 2007) systems through altering the function of GABAergic interneurons and the balance of excitation and inhibition, thus increasing glutamatergic input in areas such as the NAc or PFC (Karreman & Moghaddam, 1996; Moorman et al., 2015). In this study, we chose to examine three distinct GABAergic interneuron populations: neuronal nitric oxide synthase-expressing interneurons, parvalbumin-expressing interneurons and calretinin-expressing interneurons.

Neuronal nitric oxide synthase (nNOS) is an enzyme responsible for production of the free radical nitric oxide (NO), a potent signalling molecule that has been implicated in physiological processes such as inflammation and synaptic plasticity (Bredt & Snyder, 1992; Cadet & Brannock, 1998; Garthwaite, 2019; Hardingham et al., 2013). Functionally, nNOS-expressing GABAergic interneurons are thought to regulate non-synaptic GABA release (Le Roux et al., 2009), stimulating long-lasting hyperpolarisation and inactivation of local circuits (Taniguchi et al., 2011). Drugs of abuse have been hypothesised to increase production of neurotoxic NO, which may penetrate corticostriatal glutamatergic terminals and trigger excitotoxic effects in glutamate systems (Cadet & Brannock, 1998; Deng & Cadet, 1999). Nitric oxide has been implicated in the behavioural effects induced by METH, morphine and cocaine (Itzhak, 1997; Itzhak et al., 1998; Smith et al., 2017; Zarrindast et al., 2003). Findings from Deng and Cadet (1999) have provided evidence of increased nNOS expression at 1 h and 24 h post-acute METH exposure. The long-term effects on nitrergic systems following more prolonged exposure to METH are unknown. It is plausible that increased activation of nNOS interneurons within the PFC or NAc could enhance the overall production of NO and contribute to METH-induced neurotoxicity (Deng & Cadet, 1999; Fricks-Gleason & Keefe, 2013; Hardingham et al., 2013).

Many GABAergic interneuron populations are found to express calcium binding proteins, including parvalbumin, calretinin and calbindin, with some populations co-expressing multiple proteins or enzymes (Kubota et al., 1994). Calcium binding proteins are thought to be involved in mediating neurotoxic insult (Mura et al., 2000), and thus expression may change following exposure to METH. In the present study, we have examined two GABAergic interneuron populations expressing calcium binding proteins: parvalbumin-expressing and calretinin-expressing interneurons.

Parvalbumin (PV) is expressed in approximately 40% of all GABAergic neurons within the neocortex (Rudy et al., 2011; Tremblay et al., 2016). PV-expressing interneurons are responsible for generating network oscillations and modulating the activity of pyramidal cells and inhibitory subtypes via feedback and feed-forward mechanisms, contributing to the maintenance of the excitatory/inhibitory balance (Ferguson & Gao, 2018). Previous research has revealed both downregulation and upregulation of PV expression in the PFC following exposure to psychostimulants (Mohila & Onn, 2005; Todtenkopf et al., 2004; Veerasakul et al., 2016; Wearne et al., 2015). Considering PV interneurons are known to modulate temporal control by directly synapsing onto glutamatergic pyramidal cells (Ferguson & Gao, 2018), METH-induced dysfunction or loss of PV interneurons could contribute to increased glutamatergic outflow from the PFC to NAc observed following exposure to METH.

Calretinin is expressed in a subset of GABAergic interneurons in the mammalian cortex, accounting for approximately 30% of all GABAergic interneurons (Cauli et al., 2014; Kubota et al., 1994). Calretinin is thought to provide a neuroprotective role in neurons, buffering against calcium overload and excitotoxicity (D'Orlando et al., 2002; Lukas & Jones, 1994; Mura et al., 2000). However, calretinin-expressing interneurons may also be vulnerable to excitotoxicity themselves (Clark et al., 2017; Jászai et al., 1998; Möckel & Fischer, 1994; Tóth et al., 2010), and thus exposure to METH may impair calretinin function. Indeed, administration of METH has previously been reported to decrease calretinin expression within the frontal cortex (Veerasakul et al., 2016).

Whilst previous studies have explored the effects of acute METH on GABAergic interneuron populations, drug-induced neuroadaptations are highly dependent on factors such as the amount of drug consumed, contingency of drug administration and further influenced by periods of withdrawal, abstinence or incubation (Yager et al., 2015). Thus, it is important to investigate how chronic METH self-administration and reinstatement may affect GABAergic interneuron populations in the NAc, PFC and OFC, as changes to these cell types may contribute to neurotoxicity and impaired inhibitory control. A secondary aim of this study was to determine how METH self-administration and reinstatement alter functional connectivity between the NAc, PFC and OFC. Enhanced knowledge of the neurobiological mechanisms underlying addiction, particularly how METH may disrupt the GABA system, may yield new insights into potential treatment strategies for METH use disorder. We hypothesise that exposure to self-administered METH may alter the number or activity of GABAergic neurons in the NAc, PFC and OFC, subsequently altering the balance of excitation and inhibition, and contributing to the pathogenesis of addiction. Lack of understanding of the neurobiological mechanisms underpinning METH use disorder hinders the development of effective pharmacotherapies; thus, it is critical to gain new insight into the neurobiological mechanisms that underlie METH use disorder for the purpose of developing new treatments.

## Materials and methods

### Animals

Thirty-two male Long Evans rats (250–290 g) were obtained from the Australian Resources Centre (Perth, WA, Australia). Rats were pair-housed (cage size: 40 × 27 × 16 cm) for the entirety of the experiment excluding 2 days of individual housing post-surgery. Rats were maintained in a temperature and light-controlled room (21 ± 1 °C, 12-h light/dark cycle) and all experiments were conducted during the light cycle. Rats were provided environmental enrichment, and food and water were available *ad libitum* in home cages. Rats were acclimated for 7 days and were handled daily by the experimenter for an additional 5 days prior to surgery. All experimental procedures were conducted in accordance with the Australian Code of Practice for the Care and Use of Animals for Scientific Purposes (8th Edition, 2013) and were approved by the Macquarie University Animal Ethics Committee (ARA: 2017/043).

### Surgery

Rats underwent surgery to implant chronic indwelling catheters into the right jugular vein. Rats were anaesthetised with 3% isoflurane in oxygen (2 L/min) and were administered analgesic carprofen (Cenvet, Kings Park, NSW, 5 mg/kg, SC) and 0.9% saline (2 mL, SC). Catheters were implanted using an aseptic surgical technique and were flushed with 0.2 mL of antibiotic cephazolin sodium (20 mg/mL) immediately following implantation. Catheter construction and implantation were performed as previously described (Everett et al., 2018). Post-surgery, rats were returned to individual housing for 2 days where carprofen and saline were administered daily (SC).

### Drugs

Methamphetamine hydrochloride (METH, 99% purity) powder was purchased from Australian Government Analytical Laboratories (Pymble, NSW, Australia). METH was dissolved in 0.9% saline for treatment administration. Saline administration consisted of 0.9% saline solution. For IVSA experiments, animals were administered 0.1 mg/kg/infusion of METH or saline. For drug-primed reinstatement, rats were intraperitoneally injected (IP) with 1 mg/kg of METH or saline (1 mL/kg).

### Self-administration apparatus

Behavioural testing was conducted in 16 standard operant response chambers (32 × 25 × 34 cm: Med Associates, St Albans, VT, USA). Operant response chambers were housed in sound-attenuating boxes (41 × 56 × 56 cm) equipped with a fan for ventilation. Each chamber was equipped with two retractable levers, two cue lights and a house light. Chambers also contained a metal arm and a spring connector which was attached to the animal’s catheter. Polyethylene tubing was attached to a 10-mL syringe, driven by an infusion pump (Med Associates). Four infrared detectors were used to measure locomotor activity during test sessions. Active and inactive lever presses, number of infusions and locomotor activity were recorded using MED-PC software (Med Associates).

### Acquisition and maintenance of METH self-administration

Rats were randomly allocated to one of two IVSA groups; rats were either able to lever press for METH (*n* = 22) or were designated as yoked saline controls (*n* = 10). Rats acquired self-administration during 3-h fixed ratio 1 (FR1) scheduled sessions conducted 5 days a week for a total of 20 days. Levers were allocated as active or inactive, where the location of active lever was counterbalanced across chambers and groups. Prior to the commencement of each intravenous self-administration (IVSA) session, catheters were flushed with 0.1 mL of heparinised saline (10 IU/mL). Rats were then placed inside the operant chamber and the IVSA session was initiated by lever extension and house light illumination. Depression of the active lever resulted in a 3-s infusion of METH (0.1 mg/kg/0.05 ml infusion) or saline, which was signalled by 3-s illumination of the cue light positioned above the active lever. During the infusion, the house light was extinguished, and a 20-s time-out period occurred, during which depression of the levers was recorded without consequence. Depression of the inactive lever had no programmed consequences. Rats were limited to a maximum of 100 infusions per 3-h session (10 mg/kg of METH, IV). Any animals that did not display a preference for the drug-paired lever at the conclusion of the 20-day IVSA period were excluded from further analysis.

### Extinction of METH self-administration

Following the acquisition of METH IVSA, rats were exposed to daily sessions to extinguish the operant association between lever pressing and drug reward. These sessions decreased in length progressively (days 1–3: 180 min, days 4–7: 120 min, days 8–14: 90 min), and were identical to IVSA sessions except no drug was delivered upon lever press. That is, depression of the active lever triggered 3-s illumination of the cue light and a time-out period, as signalled by elimination of the house light (20-s); however, no infusion was delivered. On the final day of extinction, rats were administered 1 mL/kg of saline IP to habituate to drug-primed reinstatement injections. The extinction period extended for 14–15 days until active lever pressing was < 30% of responding during METH IVSA.

### Reinstatement of METH self-administration

Rats underwent one reinstatement test and a between-subjects design was employed (2 × 2); animals were injected with either saline (1 mL/kg) or METH (1 mg/kg, IP) then placed into operant response chambers for a total of 90 min. This design enabled investigation of four groups: saline IVSA and saline prime (Sal/Sal; *n* = 5), saline IVSA and METH-prime (Sal/METH; *n* = 5), METH IVSA and saline prime (METH/Sal; *n* = 8) and METH IVSA and METH-prime (METH/METH; *n* = 8). METH intake and active and inactive lever pressing behaviour did not differ across METH IVSA groups. Depression of the active lever during reinstatement sessions had no programmed consequences.

### Euthanasia and histology

Upon the completion of the reinstatement session, rats were heavily anaesthetised via an injection of sodium pentobarbitone (135 mg/2 mL, IP) and underwent intracardiac perfusion with ice cold Dulbecco’s Modified Eagle’s Medium/Nutrient Mixture F-12 Ham followed by 4% paraformaldehyde (PFA). Brains were removed and post-fixed in 4% PFA overnight at 4 °C. Brains were then placed into graded sucrose solutions and cryopreserved. Brains were sectioned coronally (40 µm thick, 1:5 serial sections) using a vibrating microtome (VTS1200S; Leica Microsystems). Free-floating brain sections were incubated in 50% ethanol for antigen retrieval and blocked in 5% normal horse serum (NHS) diluted in 0.1 M phosphate-buffered saline with 0.3% Tris and 0.05% merthiolate (TPBSm) for 2-h at room temperature to prevent non-specific binding. Primary antibodies (1:1000 goat anti-Fos, catalogue sc-52-G, Santa Cruz; 1:1000 mouse anti-parvalbumin, catalogue P3088, Sigma; 1:500 rabbit anti-nNOS, catalogue SAB4502010, Sigma; 1:1000 rabbit anti-calretinin, catalogue C7479, Sigma) were diluted in TPBSm with 5% NHS and applied for 68-h. Fluorescent conjugated secondary antibodies (Jackson ImmunoResearch) were diluted 1:500 in 2% NHS in TPBSm and applied for 24-h. Sections were mounted onto glass slides and coverslipped with Dako fluorescence mounting media (Agilent, Mulgrave, Victoria, Australia).

### Image analysis and quantification

**Fig. 1:**
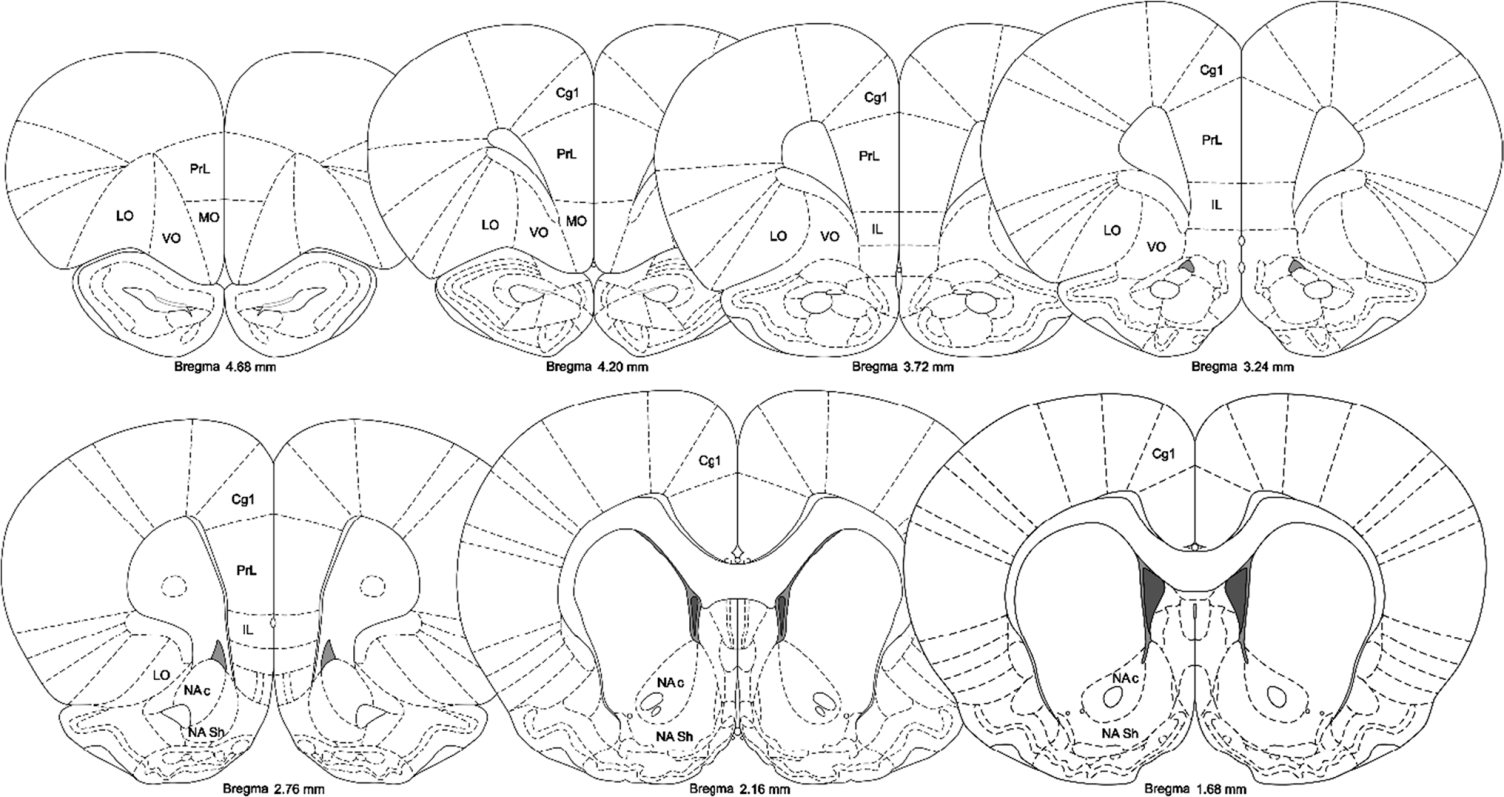
Anatomical coronal diagrams depicting each the brain region of interest and the coordinates (mm from bregma) of each subregion analysed; NAc, nucleus accumbens core; NA Sh, nucleus accumbens shell; Cg1, anterior cingulate gyrus; PrL, prelimbic cortex; IL, infralimbic cortex; MO, medial orbitofrontal cortex; VO, ventral orbitofrontal cortex; LO, lateral orbitofrontal cortex. Adapted from the Rat Brain Atlas (Paxinos and Watson, 2007)

A total of five brains per group were used for immunohistochemistry, with one brain hemisphere randomly analysed from each animal. Of the eight animals allocated to the METH/METH group, the five animals with highest number of active lever presses during reinstatement were selected as these animals were considered most likely to evidence relapse-induced neurochemical changes. Within the NAc, PFC and OFC, a total of eight subregions of interest (ROIs) were analysed across seven equidistant coordinates (ranging from + 1.7 to + 4.7 mm from bregma; Fig. 1). These subregions include the core and shell of the NAc, cingulate gyrus (Cg1), prelimbic cortex (PrL), infralimbic cortex (IL) subregions of the medial PFC and lateral, ventral and medial subregions of the OFC. Sections were imaged under epifluorescence (Zeiss AxioImager Z2 microscope 10 × /0.30M27 objective lens running ZEN 2011) at consistent exposure times. Tiled images were captured at 10 × magnification, stitched and adjusted for brightness and contrast in an identical manner using Zen software (Zeiss, Gottingen, Germany). The total number of positive immunoreactive neurons within each ROI was manually counted by an experimenter blind to treatment conditions using ImageJ (Schindelin et al., 2012). Furthermore, Fos immunoreactivity was cross-correlated between brain regions, providing an indication of which brain pathways were functionally connected, differentially between groups (Castilla-Ortega et al., 2016, 2014). This approach was also used to analyse the relationship between immunoreactivity and total METH intake.

### Statistical analyses

To determine if rats had acquired METH self-administration, we compared lever pressing, infusions and locomotor activity across the IVSA period using a repeated measures ANOVA, with the first and final day directly compared. Paired *t*-tests were used to analyse the number of active and inactive lever presses on the final day of IVSA (within METH/Sal and METH/METH groups) and extinction of pressing the drug-paired lever (comparing the mean number of presses during the final 3 days of extinction to the final 3 days of IVSA). Paired *t*-tests were also used to compare active lever presses and METH intake (mg/kg) across METH/Sal and METH/METH groups. To determine if METH/METH-treated rats had reinstated to previous METH-paired lever pressing, the total number of active lever presses during reinstatement was compared across all reinstatement conditions and to the extinction day prior using a one-way ANOVA. Total locomotor activity was also compared across all reinstatement conditions using a one-way ANOVA. The total number of immunoreactive neurons were analysed using a two-way ANOVA (factor 1: IVSA treatment, factor 2: priming treatment during reinstatement). Pearson’s correlation coefficients were used to investigate any relationship between METH intake and total number of immunoreactive neurons. For correlational analysis, both *p* and *q* values were calculated to control for a 5% false discovery rate (Storey, 2002). All statistical analyses were performed using SPSS version 20 for PC (SPSS Incorporated, Chicago, IL, USA). Data are presented as mean ± SEM. Differences were considered significant when *p* < 0.05.

## Results

### Excluded animals

Of the original 32 animals that underwent intravenous catheter implantation, seven animals were excluded due to failure to acquire METH IVSA (*n* = 2), failure to extinguish drug-paired responding on the active lever (*n* = 4) and spontaneous reinstatement of drug-paired responding following saline prime (*n* = 1).

### Intravenous METH self-administration

**Fig. 2:**
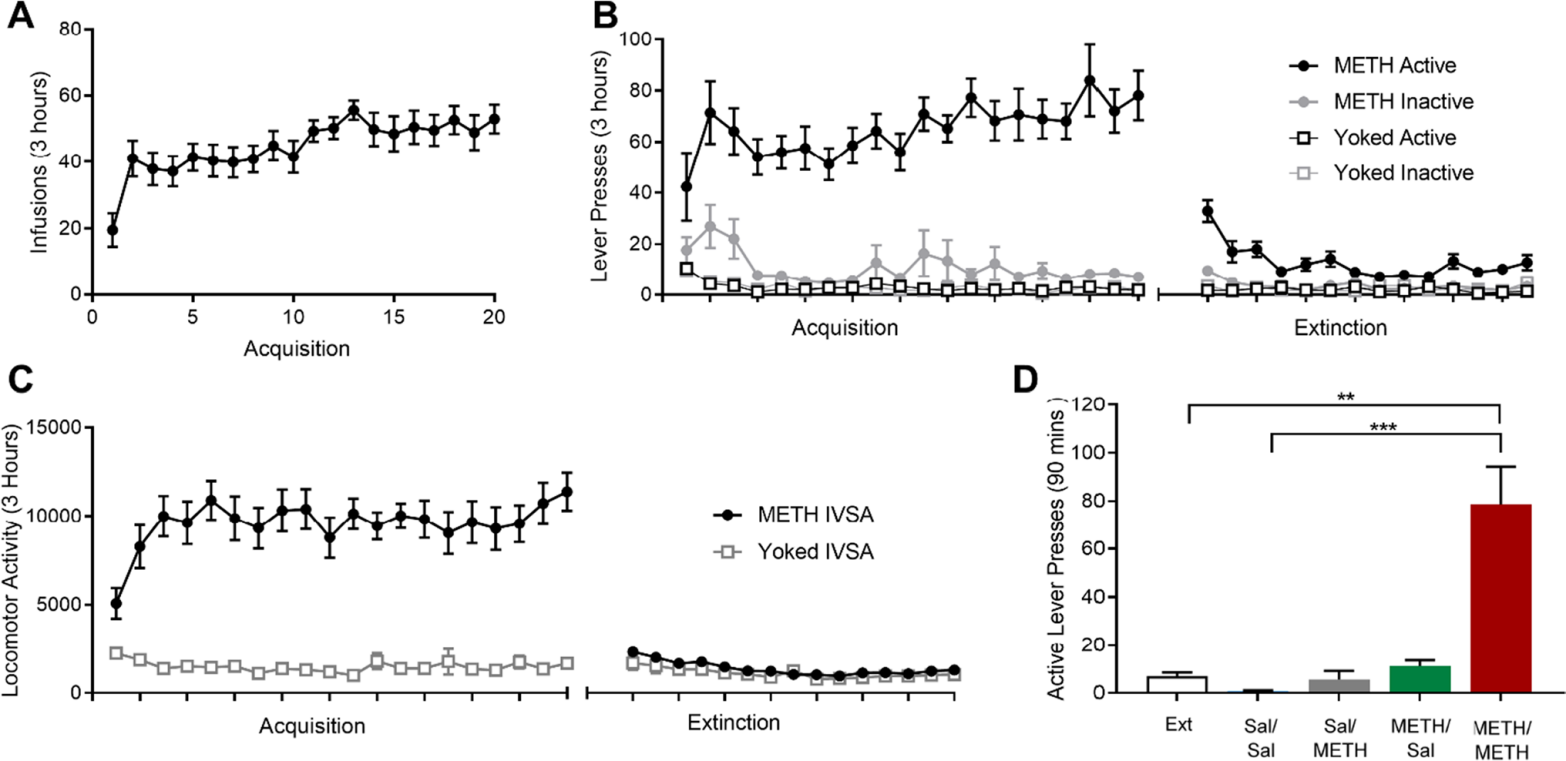
Mean number of delivered infusions (**A**), active and inactive lever presses (**B**) and locomotor activity counts (**C**) during IVSA and extinction in METH IVSA and yoked controls. Data presented as *n* = 22 METH IVSA rats, *n* = 10 yoked controls. **D** Administration of a non-contingent METH-prime significantly increased active lever pressing in METH/METH animals when compared to Sal/Sal treated animals (**p < 0.001) and the extinction day prior (**p = 0.001). All data presented as mean + SEM. Data presented as *n* = 5 in Sal/Sal and Sal/METH groups, *n* = 10 in METH/Sal and METH/METH groups

Rats acquired intravenous METH self-administration, as evidenced by a significant increase in the number of delivered infusions from day 1 to day 20 (*F*(1, 17) = 30.706, *p* = 0.000; Fig. 2A). The number of responses on the active lever also significantly increased across the IVSA period (*F*(1, 17) = 5.476, *p* = 0.032; Fig. 2B). Rats displayed a distinct preference for the drug-paired lever over the inactive lever on day 20 (*t*(16) = 8.642; *p* = 0.000). When comparing the METH/Sal and METH/METH groups, METH intake during IVSA was not significantly different (*t*(9) =  − 0.772; *p* = 0.460; Supplementary Fig. 1A). Locomotor activity was also significantly increased from day 1 to day 20 (*F*(1, 16) = 29.401, *p* = 0.000; Fig. 2C).

### Behavioural extinction

Active lever pressing was significantly reduced from the final 3 days of IVSA to the final 3 days of extinction in METH IVSA animals (*t*(29) =  − 5.630; *p* = 0.000). Additionally, similar active lever pressing behaviour was observed during the final 3 days of IVSA (*t*(9) =  − 1.127; *p* = 0.289) and the final 3 days of extinction (*t*(9) =  − 0.664; *p* = 0.523) between the two groups, suggesting that both groups had a similar METH IVSA experience.

### METH-primed reinstatement

Administration of a non-contingent METH-prime produced robust reinstatement of active lever pressing in the METH/METH animals (*F*(1, 16) = 30.964, *p* = 0.000). The number of active lever presses was significantly increased when compared to the extinction day prior (*t*(3) = 4.680, *p* = 0.001; Fig. 2D). Active lever pressing was also significantly increased in the METH/METH group when compared to inactive lever presses (*t*(9) = 5.469, *p* = 0.000; Supplementary Fig. 1B), suggesting that increased lever pressing was specific to drug-seeking behaviour and not due to METH-induced hyperlocomotion. Locomotor activity was also significantly increased in the METH/METH group when compared to other treatment conditions (*F*(1, 16) = 37.9502, *p* = 0.000) and the extinction day prior (*t*(3) =  − 3.632, *p* = 0.036; Supplementary Fig. 1C).

### METH exposure altered immunoreactivity of GABAergic interneuron populations

**Fig. 3:**
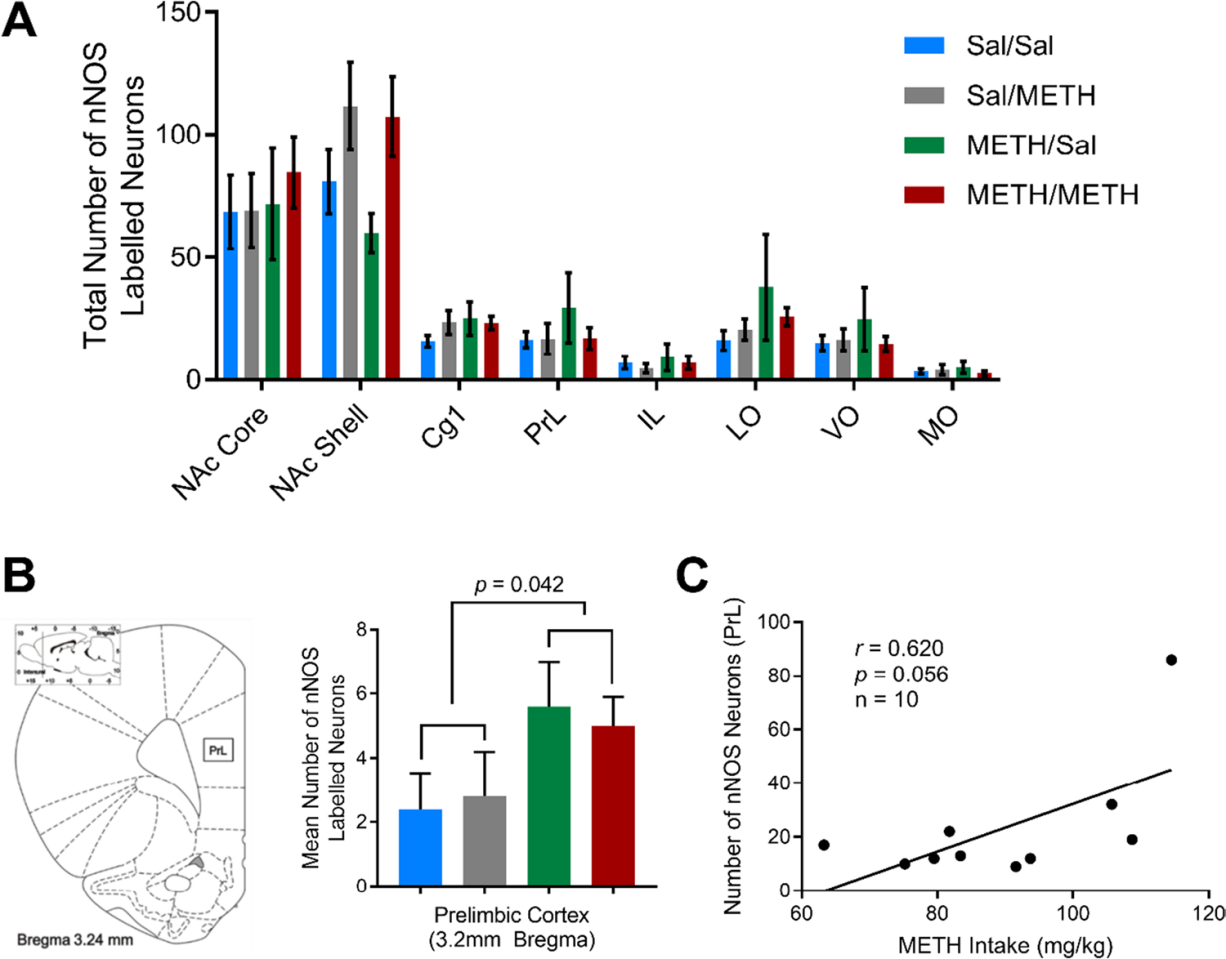
Analysis of total nNOS counts within examined brain regions revealed no significant differences across groups (**A**). Examination of nNOS reactivity in one coordinate of the PrL (+ 3.2 mm from bregma) revealed a significant effect of METH IVSA (**B**). Correlation analysis revealed a positive correlation between nNOS immunoreactivity in the PrL and METH intake, which trended towards significance (**C**). All data presented as mean + SEM. Data presented as *n* = 5 per group or *n* = 10–20 for correlational analysis

Overall, METH exposure did not significantly affect nNOS immunoreactivity in the NAc core [IVSA treatment: *F*(1, 16) = 0.301, *p* = 0.591; priming treatment: *F*(1, 16) = 0.157, *p* = 0.697; IVSA treatment × priming treatment: *F*(1, 16) = 0.604, *p* = 0.448; Fig. 3A]. However, specifically at the rostral NAc core (+ 2.2 mm from bregma), there was a moderate positive correlation between nNOS immunoreactivity and Fos immunoreactivity, which was statistically significant (*r* = 0.569, *p* = 0.011; *n* = 19; Supplementary Fig. 2A).

Upon analysis of a specific subregion of the PrL (+ 3.2 mm from bregma), METH self-administration significantly increased nNOS immunoreactivity compared with yoked-saline rats [IVSA treatment: *F*(1, 16) = 4.893, *p* = 0.042; Fig. 3B]. This effect appears to be specific to prolonged METH exposure, as there was no effect of priming treatment [*F*(1, 16) = 0.007, *p* = 0.936] and no interaction of IVSA and priming [*F*(1, 16) = 0.168, *p* = 0.688]. This finding is further supported by a positive correlation between the total number of nNOS immunoreactive neurons in the PrL and METH intake, although this was not statistically significant (*r* = 0.620, *p* = 0.056; Fig. 3C). However, when PrL coordinates were combined to enable examination of nNOS immunoreactivity across the entire PrL, there was a no effect of METH exposure on nNOS immunoreactivity [*F*(1, 16) = 0.642, *p* = 0.435].

**Fig. 4:**
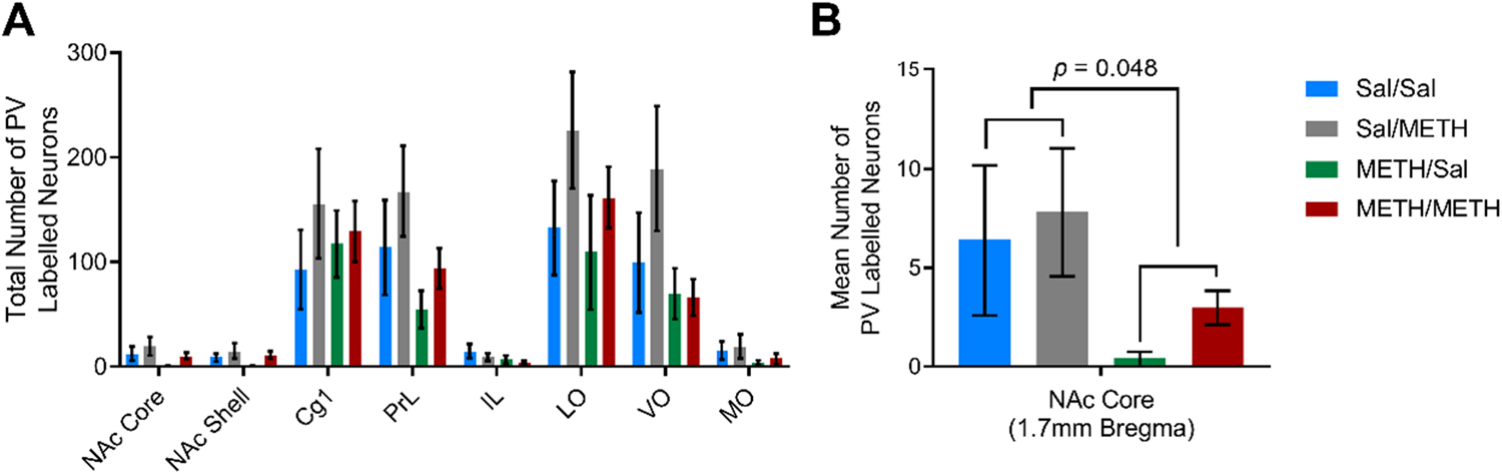
Analysis of total regional PV immunoreactivity revealed no statistically significant differences across treatment groups (**A**). However, at one discrete coordinate of the NAc core (+ 1.7 mm from bregma), METH-IVSA yielded a significant reduction in PV immunoreactivity (**B**). Data presented as mean ∓ SEM, *n* = 5 per group

In addition, we found no statistically significant effect of METH IVSA or METH priming on PV immunoreactivity in all regions analysed (Fig. 4A), except at one caudal coordinate of NAc core where we observed a modest reduction in PV immunoreactivity following METH IVSA treatment, compared with yoked-saline rats [NAc core + 1.7 mm: *F*(1, 16) = 4.563, *p* = 0.048; Fig. 4B].

**Fig. 5:**
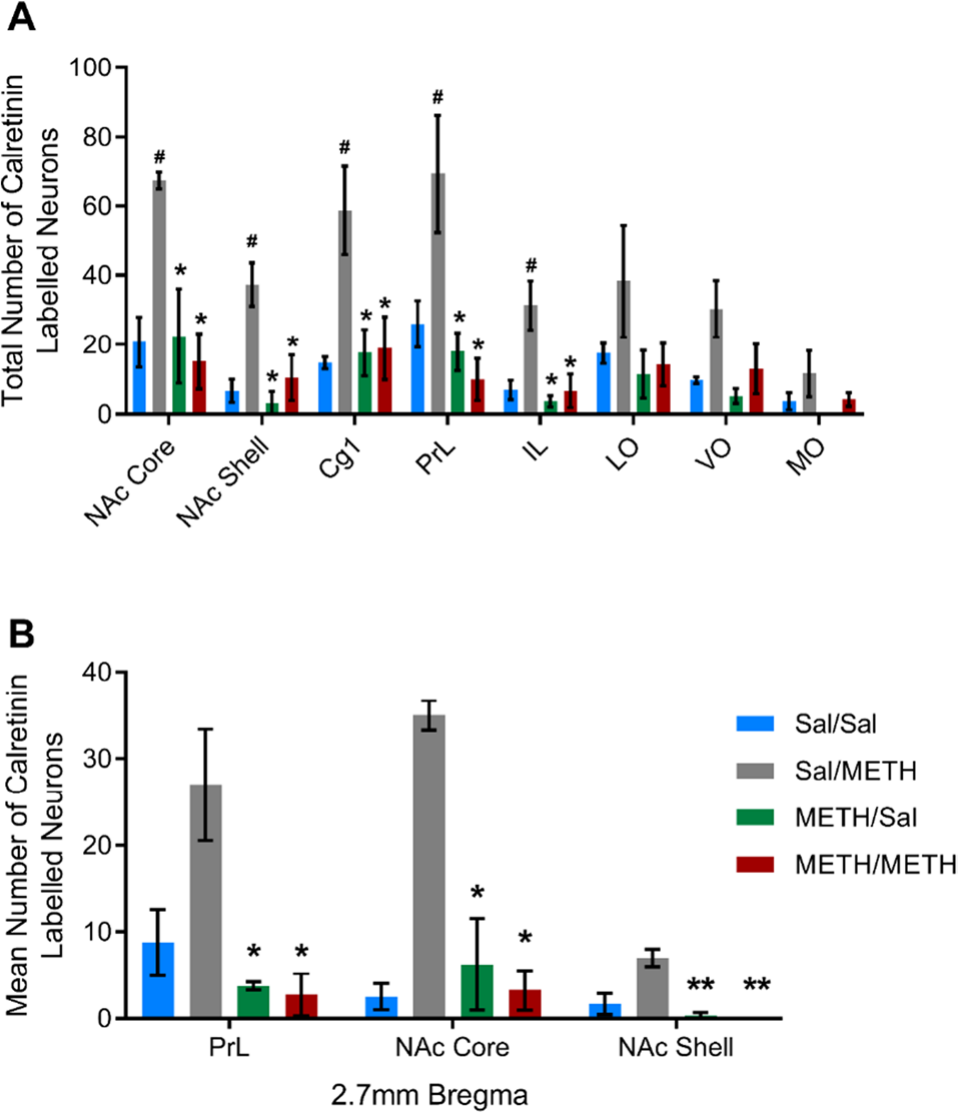
Analysis of total counts of calretinin labelled neurons across brain regions revealed a significant reduction in calretinin immunoreactivity in METH IVSA animals and a significant increase in Sal/METH-exposed animals in the NAc core, NAc shell, Cg1, PrL and IL (**A**). Examination of one specific coordinate of the brain (+2.7 mm from bregma) also revealed a significant reduction in calretinin immunoreactivity in METH IVSA animals (**B**). Data presented as mean ± SEM, *n* = 4 per group. * represents a significant effect of METH IVSA, # represents a significant interaction effect whereby Sal/METH differed to all other groups

Analysis of total calretinin counts revealed a significant effect of METH IVSA treatment on calretinin immunoreactivity, with METH self-administration producing a significant decrease in the number of calretinin immunoreactive neurons in the PrL [IVSA treatment: *F*(1, 14) = 14.351, *p* = 0.004], IL [IVSA treatment: *F*(1, 14) = 8.837, *p* = 0.014], NAc core [IVSA treatment: *F*(1, 14) = 5.315, *p* = 0.044] and NAc shell [IVSA treatment: *F*(1, 14) = 7.460, *p* = 0.021; Fig. 5A]. We also found interaction effects in the Cg1, PrL, IL, NAc core and NAc shell subregions, whereby calretinin immunoreactivity was found to be significantly increased in Sal/METH animals but decreased in METH IVSA animals [Cg1: *F*(1, 14) = 8.203, *p* = 0.017; PrL: *F*(1, 14) = 8.259, *p* = 0.017; IL: *F*(1, 14) = 6.378, *p* = 0.030; NAc core: *F*(1, 14) = 10.614, *p* = 0.009; NAc shell: *F*(1, 14) = 5.198, *p* = 0.046]. Interestingly, analysis of independent bregma coordinates revealed a significant effect of IVSA treatment at + 2.7 mm from bregma in the PrL [IVSA treatment: *F*(1, 17) = 5.315, *p* = 0.038], NAc core [IVSA treatment: *F*(1, 17) = 5.533, *p* = 0.035] and NAc shell [IVSA treatment: *F*(1, 14) = 12.511, *p* = 0.005; Fig. 5B]. Calretinin immunoreactivity in the OFC was found to be unaffected by METH exposure, with no significant effects detected in LO, VO or MO subregions.

### Administration of METH increases neuronal activity

**Fig. 6:**
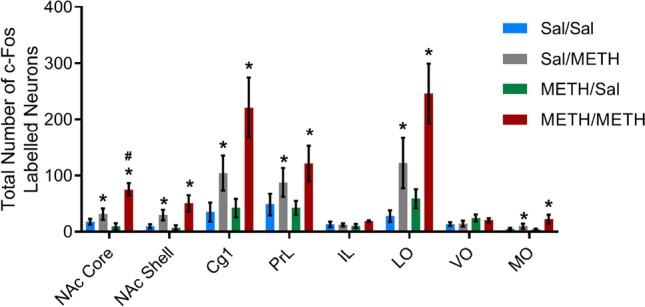
Treatment groups which received a non-contingent injection of METH (1 mg/kg) during relapse testing displayed significantly increased Fos immunoreactivity when compared to groups treated with vehicle (*p < 0.05). NAc core Fos immunoreactivity was also significantly increased in METH/METH animals when compared to Sal/METH animals (#*p* = 0.020). Data presented as mean  ∓SEM. Data presented as *n* = 4 in METH/Sal group, *n* = 5 in all other groups

We identified a main effect of METH-priming treatment on reinstatement test day, with rats that received a non-contingent injection of METH (Sal/METH and METH/METH groups) displaying increased Fos immunoreactivity in the NAc core, medial PFC and OFC [NAc core *F*(1, 15) = 20.022, *p* = 0.000; NAc shell *F*(1, 15) = 10.670, *p* = 0.005; Cg1 *F*(1, 15) = 12.923, *p* = 0.003; PrL *F*(1, 15) = 5.785, *p* = 0.030; LO *F*(1, 15) = 13.714, *p* = 0.002; MO *F*(1, 15) = 5.058, *p* = 0.040; Fig. 6]. There was no effect of METH-priming treatment or METH IVSA on Fos immunoreactivity in the infralimbic and ventral orbitofrontal cortices [IL *F*(1, 15) = 1.336, *p* = 0.266; VO *F*(1, 15) = 0.136, *p* = 0.718]. We observed a significant interaction effect between IVSA and priming treatment in the NAc core, whereby the increased NAc core activity caused by METH injection was greater in animals that self-administered METH compared to yoked saline controls [*F*(1, 15) = 8.614, *p* = 0.010]. This was accompanied by a statistically significant increase in Fos immunoreactivity in METH/METH animals when directly compared to Sal/METH animals (*t*(8) =  − 2.896, *p* = 0.020).

**Fig. 7:**
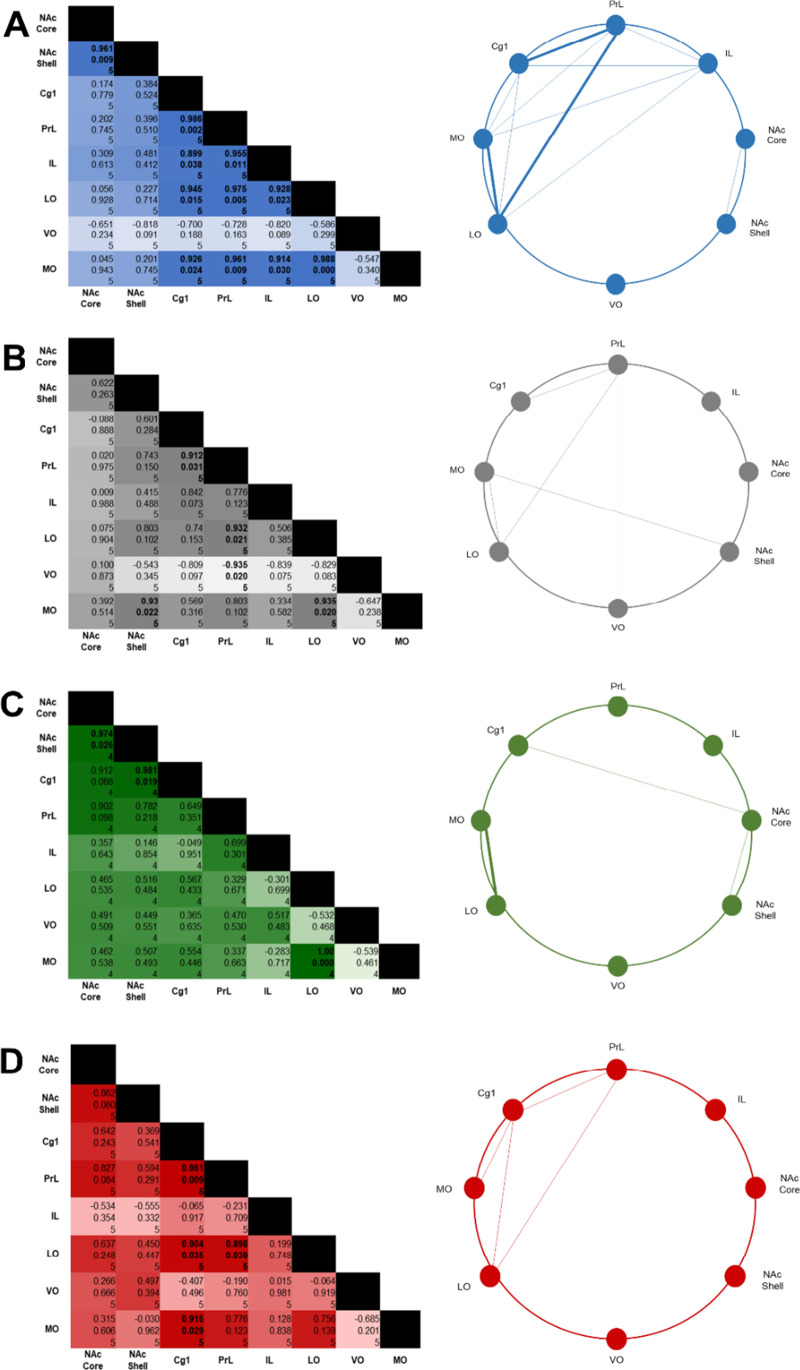
Pearson’s correlation coefficients and connectivity diagrams for Sal/Sal (**A**), Sal/METH (**B**), METH/Sal (**C**) and METH/METH (**D**) treated animals. LHS: Matrices describing the Pearson’s correlation coefficient, *p* value and *n* value. The strength of the correlation is depicted by the shade, with weak correlations presented in lighter shades and stronger correlations presented in darker shades. Statistically significant correlations are presented in bold. RHS: Diagrams displaying possible functional connectivity between examined brain regions. Solid lines represent positive correlations which were statistically significant. Bold lines represent significant positive correlations which met *q* criteria

Pearson’s correlation analysis was performed within groups to examine, firstly, if changes in neuronal activity were correlated across subregions of interest, and secondly, if these functional connections were altered by a history of METH self-administration, or by a METH injection. Correlated activity was decreased in all METH-experienced conditions compared to Sal/Sal controls, with Sal/METH and METH/METH animals displaying four significant correlations, whilst METH/Sal animals displayed three significant correlations (Fig. 7). However, many correlations did not meet *q* criteria and should be interpreted with caution (Storey, 2002).

Under Sal/Sal conditions, it appears neuronal activation in the IL is correlated with activity in the Cg1, PrL, LO and MO. Markedly, in conditions with any METH history, the IL does not appear to be functionally correlated with any other examined brain region. Furthermore, under saline priming conditions, neuronal activity in the NAc core was highly correlated with activity in the NAc shell. Conversely, Sal/METH and METH/METH rats exhibited a lack of correlated activity between subregions of the NAc. Interestingly, activation in the MO and LO was correlated in all treatment conditions except METH/METH animals.

Fos immunoreactivity was analysed for co-localisation with nNOS, PV and calretinin immunoreactivity. Of the neurons which were immunoreactive for nNOS, only a small proportion co-expressed Fos in the NAc core and PrL, suggesting minimal activation of nNOS interneurons in these brain regions across all treatment groups (Supplementary Fig. 2B). Analysis of activated nNOS neurons within the PrL revealed a trend towards a significant effect of METH IVSA treatment [*F*(1, 16) = 0.3.229, *p* = 0.092], suggesting that self-administration of METH may trigger subtle increases in nNOS activation. Analysis of co-localisation revealed that only a small proportion of Fos immunoreactivity was co-localised with PV or calretinin immunoreactivity in all examined subregions. Furthermore, there were no differences in activation of immunoreactive interneuron populations across treatment groups.

## Discussion

The primary aim of this study was to investigate how chronic METH self-administration and reinstatement may alter the number and activity of GABAergic interneuron populations, specifically parvalbumin, nNOS and calretinin-expressing interneurons, in the NAc, PFC and OFC. Identification of changes to the activity of the neuronal populations may suggest dysfunctional balance of excitation and inhibition within reward circuitry, contributing to altered inhibitory control and neurotoxicity. We hypothesised that repeated exposure to METH may cause selective dysfunction or degeneration of GABAergic interneuron populations, leading to aberrant increases in excitatory tone and neurotoxicity. The secondary aim of this study was to determine the impact of METH self-administration and reinstatement on neuronal activity within subregions of the NAc, PFC and OFC. It was anticipated that METH exposure would increase neuronal activity in reward-associated brain regions in animals exposed to METH-priming treatment, potentially leading to dysregulated activity of functional circuits.

### Exposure to METH produced differential effects on GABAergic interneuron populations

We found that chronic METH self-administration had no impact on nNOS immunoreactivity in most examined brain areas (six subregions, 7 different bregma levels), contrasting with previous published literature implicating activation of nNOS interneurons in cocaine-associated behaviours (Nasif et al., 2011; Sammut & West, 2008; Smith et al., 2017). However, analysis of one discrete coordinate of the PrL (+ 3.2 mm from bregma) did reveal a significant increase in nNOS immunoreactivity following METH IVSA. Additionally, nNOS immunoreactivity in the PrL was also positively correlated with METH intake. Collectively, these findings suggest that repeated METH exposure may be required to induce pathology within the PrL, as acute METH exposure did not produce any discernible effects on nNOS immunoreactivity in the PrL, aligning with findings from Deng and Cadet (1999) who reported no change in nNOS expression in the cortex 1-h post-METH administration. Increased expression of nNOS within the PrL may increase production of neurotoxic NO, leading to dysfunction and degeneration of monoaminergic systems within reward circuitry (Cadet & Brannock, 1998; Deng & Cadet, 1999).

Analysis of a specific coordinate of the NAc core revealed a strong positive correlation between nNOS immunoreactivity and Fos immunoreactivity which was statistically significant. It has previously been demonstrated that METH can increase the expression of nNOS within the murine striatum (Deng & Cadet, 1999), which may act to increase production of reactive oxygen species, such as NO. Activity of nNOS is thought to be tightly regulated by calcium and calmodulin signalling (Bredt & Snyder, 1990; Hanson et al., 2018). Thus, increased calcium signalling may drive increased activity of nNOS, consequently increasing production of neurotoxic NO (Bredt & Snyder, 1990; Garthwaite, 2019). NO is hypothesised to directly bind with glutamate receptors or permeate into glutamatergic terminals, causing aberrant release of glutamate and glutamate-induced excitotoxicity (Cadet & Brannock, 1998; Garthwaite, 2019). When considered together, our findings suggest that increased neuronal activation may consequently increase activation of the nitrergic system within the PFC and NAc core, causing aberrant glutamate and dopamine neurotransmission which contribute to METH-relapse behaviours and/or neurotoxic effects. These METH-induced effects on glutamate and dopamine signalling may also reinforce the rewarding properties of METH, increasing the propensity to continue METH use or reinstatement of METH-seeking behaviour after a period of abstinence.

Initially, we expected that the number or activation of PV interneurons would be altered following exposure to METH, disrupting the excitatory/inhibitory balance and causing aberrant pyramidal cell spiking (Ferguson & Gao, 2018). Unexpectedly, the results from this study revealed a non-significant effect of METH IVSA or METH-priming treatment on immunoreactivity or activation of PV interneurons. At one specific coordinate in the NAc core (+ 1.7 mm from bregma), we observed a statistically significant reduction in PV immunoreactivity in animals that were experienced in METH IVSA, aligning more closely with the findings of Veerasakul et al. (2016) who reported decreased PV immunoreactivity within the frontal cortex and hippocampus following repeated exposure to escalated doses of METH. However, this reduction in PV is relatively modest and is therefore unlikely to significantly alter overall circuit function. Interestingly, selective silencing of PV-expressing interneurons in the NAc following exposure to amphetamine inhibited the expression of a sensitised locomotor response and prevented amphetamine-induced conditioned place preference (Wang et al., 2018). These data suggest that reducing the input of PV interneurons in the NAc may attenuate drug effects, conflicting with our initial hypotheses. It is possible that PV-expressing interneurons may play a more complex, nuanced role in mediating drug reward.

In the present study, we identified a significant effect of METH self-administration on immunoreactivity of calretinin, whereby calretinin immunoreactivity was found to be decreased in the PrL, IL, NAc core and NAc shell of animals exposed to METH IVSA and METH-primed reinstatement. These findings align with initial hypotheses and previous findings by Veerasakul et al. (2016), which highlighted reduced calretinin expression in the frontal cortex following chronic exposure to METH. Prolonged exposure to METH may cause degeneration and apoptosis of calretinin-expressing GABAergic interneuron populations or loss of calretinin protein expression. Loss of calretinin-expressing interneurons could have profound functional consequences and could contribute to increased excitation via disinhibition of pyramidal cells and loss of inhibition of other inhibitory interneuron populations (Mura et al., 2000).

With acute administration of METH, there was an unexpected increase in the numbers of neurons immunoreactive for calretinin in the medial PFC (Cg1, PrL and IL) and NAc (core and shell) that was statistically significant. Calcium binding proteins are responsible for decreasing excessive calcium ions found within neurons, and transient increases in calbindin or calretinin expression have previously been reported following cytotoxin administration (Henzi et al., 2009; Huang et al., 1995; Rycerz et al., 2014). As calretinin is a calcium binding protein, it is plausible that the reported increase in calretinin immunoreactivity may reflect increased intensity or expression of the calretinin protein within calretinin-expressing interneurons. Indeed, calretinin expression has been found to be increased in the CA1 subregion of the hippocampus in rats exposed to monosodium glutamate, an experimental model of excitotoxicity (Rycerz et al., 2014). As neuron counts were conducted using intensity thresholding in the present study, increased expression of the protein may artificially increase the number of neurons that meet immunoreactivity thresholds. Thus, the reported increase in calretinin immunoreactivity may reflect increased calretinin expression following acute METH administration, a compensatory neuroprotective mechanism against increased intracellular calcium levels and glutamate-induced excitotoxicity. Previous evidence has suggested that acute dopamine depletion or degeneration of dopaminergic systems may cause a transient increase in expression of the calretinin protein within calretinin-expressing interneurons in the rodent striatum (Mura et al., 2000). Mura et al. (2000) hypothesised that dopamine depletion may alter the balance of excitation and inhibition within the striatum, leading to increased excitatory input and enhanced intracellular calcium concentrations and that calretinin expression may be increased within striatal neurons as a compensatory mechanism following dopamine depletion. As METH administration is known to cause aberrant monoamine neurotransmission and depletion of dopamine stores, thus dopamine depletion may drive transient increases in calretinin expression in acute METH-exposed animals. Interestingly, this increase in calretinin immunoreactivity was not observed in animals that had self-administered METH. This suggests that the transient increases in calretinin expression may be dampened with repeated exposure to excitotoxic stimulants such as METH, with repeated exposure to METH eventually producing loss/degeneration of calretinin neurons. The role of calretinin expression following exposure to neurotoxicants is still debated and further research is required to clarify the mechanism by which calretinin immunoreactivity is increased following acute METH but decreased following repeated exposures to METH.

### METH self-administration and reinstatement altered functional connectivity in PFC-PFC and PFC-NAc circuits

Here we have demonstrated increased neuronal activation in the NAc core, NAc shell, Cg1, PrL, LO and MO following METH-priming treatment, aligning with existing published literature (Breiter et al., 1997; Morshedi & Meredith, 2007; Volkow et al., 2012). No changes to neuronal activation were observed in the IL or VO cortices of any treatment group. Similarities in the activation patterns of the IL and VO are not surprising in light of findings by Zimmermann et al. (2018), which suggest functionality of the rodent ventrolateral OFC and IL may be closely linked.

Interestingly, we noted increased Fos reactivity in the NAc core of METH/METH compared to Sal/METH animals. This suggests that whilst acute METH administration increases neuronal activity in the NAc core, prior exposure to METH produces a more sensitised effect during METH-primed reinstatement. Scofield et al. (2016) hypothesised that relapse to drug-seeking behaviour, and the resulting increases in NAc core neuronal activity, may be driven by drug-induced plasticity of PrL-NAc core glutamatergic projections (O'Brien & Kalivas, 2008). Indeed, evidence suggests activation of glutamatergic PrL-NAc projections are critical for reinstatement of cocaine seeking (McFarland et al., 2004; McFarland et al., 2003; Scofield et al., 2016), as inhibition of this pathway has been shown to attenuate reinstatement of drug-seeking behaviour (Kearns et al., 2018; McFarland et al., 2003; Stefanik et al., 2013).

On examination of colocalisation of Fos with three GABAergic markers, nNOS, PV and calretinin, only a small proportion of the activated neurons were colocalised with GABAergic markers. Additionally, the relative proportion of nNOS, PV and calretinin neurons which were activated did not change across treatment groups. Whilst the findings of the present study suggest an overall lack of activation of GABAergic interneurons following METH-primed relapse, it is still plausible that GABAergic interneurons may interact with glutamatergic PrL-NAc core projections in a dysfunctional way, thus driving and mediating relapse to METH.

Existing neuroimaging studies conducted on cocaine and amphetamine users have highlighted disrupted functional relationships between mPFC, OFC and NAc (Breiter et al., 1997; Ersche et al., 2005; Goldstein et al., 2007); however, there is a distinct lack of research examining how METH exposure may alter functional connectivity. In the current study, we examined if changes in neuronal activity could be correlated across subregions of interest, and if this relationship was altered by METH exposure. We detected many statistically significant correlations between Fos immunoreactivity in examined subregions; however, many correlations did not meet *q* criteria (false discovery rate) and thus should be interpreted with caution. Of particular note, we observed a distinct lack of correlated neuronal activity in the LO and MO of animals that underwent METH-primed relapse, a correlation that was observed in all other treatment groups. This particular correlation was found to be robust, meeting *q* criteria and thus unlikely to be a false discovery. This suggests that functional connectivity between these two regions may be lost or impaired during relapse. Under normal conditions, the LO and MO subregions are densely interconnected and are postulated to work together to determine perceived value and expected outcomes (Lopatina et al., 2017). Lopatina et al. (2017) demonstrated that the LO utilises contextual information about tasks and behaviours, i.e. behaviour X is required to achieve Y reward, whilst the MO acts to group behavioural tasks by type, using episodic memory to place a perceived value. Hence, it is possible disrupted functional connectivity between the LO and MO may lead to dysfunctional encoding of the value of a behavioural task, consequently causing stimuli, tasks or behaviours to be overvalued. It is striking then that this loss of connectivity was found in the group undergoing METH-primed reinstatement of drug-seeking, and not in rats simply exposed to METH, suggesting that the MO-LO pathway is specifically involved in drug-seeking, and may contribute to the compulsive drive to seek METH at the expense of other stimuli or behaviours.

Our data indicates that the MO-LO pathway loses connectivity during reinstatement but is still intact during acute METH exposure and following chronic METH exposure, this suggests that MO-LO activity may follow progression of METH-induced pathology. Future studies could utilise pharmacological or chemogenetic strategies to firstly increase neuronal activity within the LO (further disrupting functional connectivity between the LO and MO subregions) and investigate if this increases reinstatement of prior drug-taking behaviour. In contrast, future experiments could investigate if pharmacological or chemogenetic inhibition of the LO subregion during METH-priming injections acts to attenuate reinstatement of prior drug-taking behaviour.

It is important to note that both contralateral and ipsilateral projections from the cortex to the striatum have been implicated in reinstatement of drug-seeking behaviours; however, these projections may play different functional roles and thus display differing levels of neuronal activity during reinstatement (James et al., 2018; McGlinchy et al., 2016). As the present study analysed only one hemisphere of the brain and did not differentiate between the left or right hemisphere, we cannot interpret how contralateral vs ipsilateral projections may influence the detected changes in neuronal activity and correlations across subregions of interest. Future studies should differentiate the left and right hemispheres, allowing examination of if neuronal activity was correlated across both contralateral and ipsilateral subregions and how this relationship may change following exposure to METH.

### Future directions and conclusions

The brain regions investigated in the current study are highly heterogeneous and express multiple subpopulations of GABAergic interneurons. Here, nNOS, PV and calretinin-expressing interneurons were investigated due to their functional role in maintaining the excitatory/inhibitory balance and controlling the output of neuronal circuits through feed-forward inhibition and temporal control of pyramidal cell firing (Ferguson & Gao, 2018; Mura et al., 2000; Pouille & Scanziani, 2001; Wehr & Zador, 2003). Whilst the methods used in this study failed to detect changes in activity of GABAergic interneurons, METH may still influence interneuron function through altering protein or RNA expression. Previous research by our group has suggested that METH-induced behavioural sensitisation can alter the PFC proteome and mRNA expression within GABAergic interneurons (Wearne et al., 2015; Wearne, Parker, Franklin, Goodchild, & Cornish, 2016). It is also plausible that whilst activity of GABAergic interneurons was unchanged in the present study, METH may change the pattern of neuronal firing or alter structural connectivity, consequently changing the activity of other neuronal populations within functional networks. Future research should aim to clarify if there are changes in electrophysiological properties or synaptic connectivity of GABAergic interneurons and investigate METH-induced changes to other GABAergic subpopulations, such as somatostatin and calbindin-expressing interneurons. Future studies should also explore whether the changes to GABAergic interneuron populations described in this study are specific to METH or whether similar changes would be observed following exposure or self-administration of a natural reinforcer.

Many factors influence the neurobiological neuroadaptations induced by drug exposure, including the amount of drug consumed, contingency of drug administration and withdrawal influences (Yager et al., 2015). The findings from the present study highlight more pronounced effects on nNOS expression following self-administration of METH, whilst acute METH was not found to produce the same effect. Therefore, it is plausible that more pronounced effects to the nitrergic system may be observed following a longer period of continued exposure or consumption of higher doses of METH. Indeed, Schwendt et al. (2009) revealed more pronounced cognitive deficits and increased evidence of METH-induced neurotoxicity in animals that were provided extended access to METH (6-h sessions) compared with animals that were provided limited access (2-h sessions). Thus, overt neurotoxicity resulting in altered activation of GABAergic interneurons may be more pronounced following escalated or binge-like patterns of METH intake. In regard to withdrawal influences, in this study animals underwent a period of forced abstinence from drug taking but were continually exposed to the drug-taking environment (IVSA chambers). Future studies could employ a similar experimental design and investigate neuronal activation and GABAergic interneuron populations during METH-primed reinstatement following a period of forced abstinence (removal from drug-taking environment and cues), or voluntary abstinence through choice for alternative social or food reinforcers (Venniro et al., 2018) rather than behavioural extinction of METH cues. Altering the study design to more closely mimic how humans abstain and relapse to drug-taking behaviour may reveal different insights about the neurobiological changes underpinning METH relapse.

In summary, this study investigated correlated neuronal activity and GABA subpopulation expression following exposure to METH. The results indicate exposure to METH produced distinct effects on GABAergic interneuron populations, with METH IVSA producing increased nNOS immunoreactivity in one coordinate of the PrL and decreased PV immunoreactivity in one coordinate of the NAc. Calretinin-expressing interneuron populations were also affected, with METH IVSA found to decrease the number of calretinin interneurons, whilst acute METH caused an increase in the number of calretinin interneurons detected. Interestingly, no changes were detected in the activation of GABAergic interneuron populations following different exposures to METH. The lack of activation of nNOS and PV interneuron populations following METH-primed relapse suggests that METH may alter GABAergic interneuron systems in ways that have not been examined in this study. Thus, whilst the present study did not detect changes in the activity of GABAergic interneuron types examined here, further research is required to comprehend the overall impact of METH exposure on the function of these heterogenous neuronal populations. Additionally, we present new insights into altered neuronal activity, importantly identifying possible impairment to LO-MO connections in METH-relapse animals. It is imperative to continue to enhance knowledge of the neurobiological mechanisms driving METH addiction, as further identification of METH-induced neuroadaptations may lead to the development of more effective pharmacotherapies for METH addiction and associated disorders.

## Supplementary Information

Below is the link to the electronic supplementary material.

Supplementary file1 (DOCX 393 KB)

## Abbreviations

ANOVA: Analysis of variance
Cg1: Anterior cingulate cortex
FR1: Fixed ratio 1 schedule
GABA: γ-Amino-butyric acid
IL: Infralimbic cortex
IP: Intraperitoneal
IV: Intravenous
IVSA: Intravenous self-administration
LO: Lateral orbitofrontal cortex
METH: Methamphetamine
MO: Medial orbitofrontal cortex
mPFC: Medial prefrontal cortex
NAc: Nucleus accumbens
nNOS: Neuronal nitric oxide synthase
NO: Nitric oxide
OFC: Orbitofrontal cortex
PFC: Prefrontal cortex
PrL: Prelimbic cortex
PV: Parvalbumin
ROI: Region of interest
Sal: Saline
SC: Subcutaneous
VO: Ventral orbitofrontal cortex
