# Supplementary material for: The effect of self-administered methamphetamine on GABAergic interneuron populations and functional connectivity of the nucleus accumbens and prefrontal cortex
Source: Psychopharmacology (Berl). 2022 Aug 3;239(9):2903–19. doi: 10.1007/s00213-022-06175-9 (PMC9385811; doi:10.1007/s00213-022-06175-9)
Supplement: Supplementary file 1 — Supplementary file1 (DOCX 393 KB) [file 213_2022_6175_MOESM1_ESM.docx]

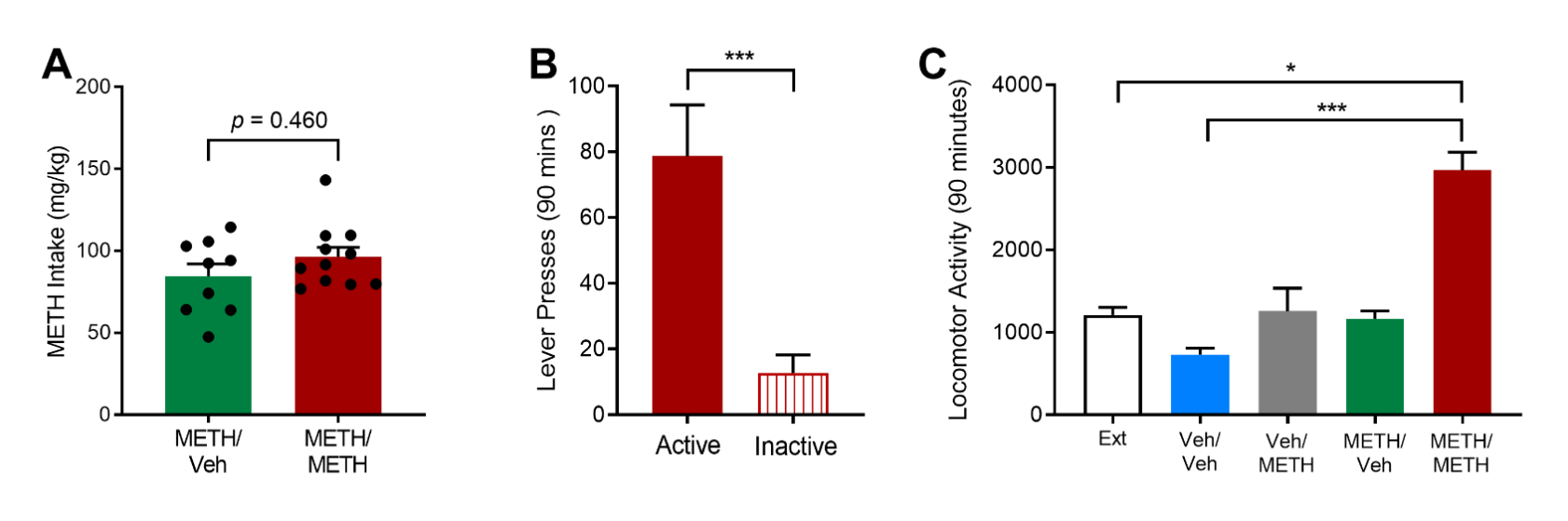


METH/
SAL

METH/
METH

Active

Inactive

METH/
METH

METH/
Sal

Sal/
METH

Sal/
Sal

Ext

Supplementary Figure 1. Total METH intake did not significantly differ between METH/Sal and METH/METH designated animals (A). In the METH/METH group, active lever pressing was significantly increased when compared to inactive lever pressing following administration of a non-contingent METH prime (B). Locomotor activity was also significant increased in METH/METH animals when compared to Sal/Sal animals and the extinction day prior (C). * represents p = 0.036, *** represents p = 0.001. All data presented as mean +SEM. Data presented as n = 5 in Sal/Sal and Sal/METH groups, n = 10 in METH/Sal and METH/METH groups.


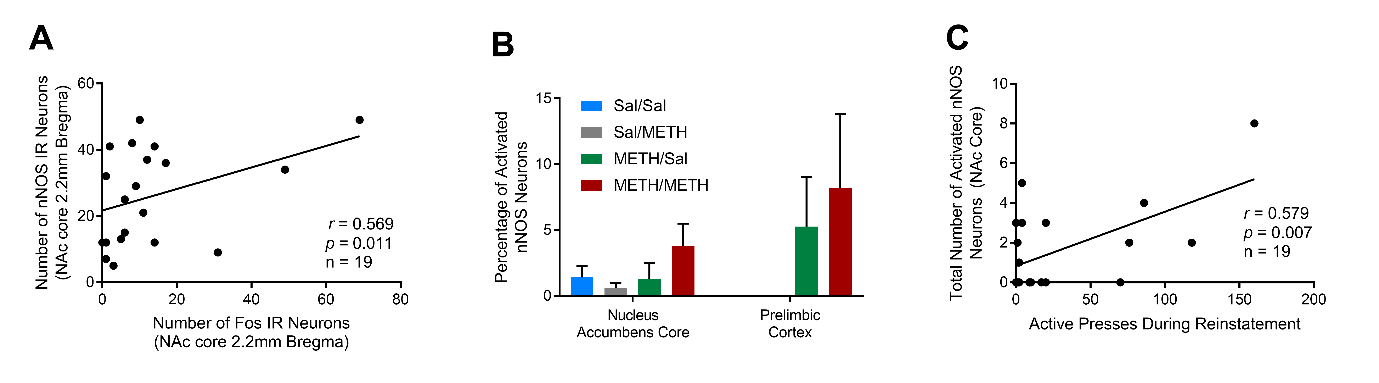


Supplementary Figure 2. Correlational analysis revealed a positive correlation between nNOS immunoreactivity in the nucleus accumbens core (+ 2.2mm from Bregma) and Fos immunoreactivity in the nucleus accumbens core which was statistically significant (A). We found no statistically significant differences across treatment groups in the percentage of nNOS immunoreactive neurons which co-expressed Fos in the nucleus accumbens core or prelimbic cortex, suggesting no differences in the pattern of activation of nNOS-expressing neurons (B). Data presented as mean + SEM.
